# Pre-diagnostic faecal calprotectin levels in patients with colorectal cancer: a retrospective study

**DOI:** 10.1186/s12885-022-09440-4

**Published:** 2022-03-24

**Authors:** Nathalie Blad, Richard Palmqvist, Pontus Karling

**Affiliations:** 1 Department of Public Health and Clinical Medicine/Medicine, Umeå University, S90185 Umeå, Sweden; 2 Department of Medical Biosciences/ Pathology, Umeå University, Umeå, Sweden

**Keywords:** Calprotectin, Colorectal cancer, Rectal cancer, Tumor localization, Tumor stage

## Abstract

**Background:**

Faecal calprotectin (FC) is a potential biomarker for colorectal cancer (CRC) screening. There is uncertainty if tumor characteristics are associated with FC levels. We investigated how tumor stage and tumor localization influence the extent of FC levels in patients with CRC in clinical practice.

**Methods:**

In two cohorts of patients with CRC, we retrospectively analyzed FC tests (CALPRO®) performed within three months prior to diagnosis. One hundred twenty-four patients with CRC were included (mean age 68 years, 44% women).

**Results:**

Ninety-eight patients with CRC (79%) had a FC ≥ 50 µg/g. FC correlated positively with tumor stage (UICC based on WHO TNM classification) (r_s_ 0.24; *p* = 0.007) and with CRP levels (r_s_ 0.31, *p* = 001), and a negatively with B-haemoglobin (r_s_ -0.21; *p* = 0.019). The patients with right-sided CRC had significantly more often a FC ≥ 50 µg/g than patients with left-sided CRC (92% vs 74% p = 0.027). In a binary logistic regression analysis, tumor stage III/IV (adjusted OR 3.47; CI 1.27–9.42) and right-sided tumor localization (adjusted OR 3.80; CI 1.01–14.3) were associated with FC ≥ 50 µg/g. Tumor stage III/IV (adjusted OR 2.30; CI 1.04–5.10) and acetylsalicylic use (adjusted OR 3.54; CI 1.03–12.2) were associated with FC ≥ 100 µg/g. In a cox regression analysis, a FC ≥ 100 µg/g was not associated with survival (Hazard OR 0.61; CI 0.24–1.52).

**Conclusions:**

Elevated pre-diagnostic FC levels were common in patients with CRC in close proximity to diagnosis. Right-sided localization and tumor stage were significantly associated with a rise in FC levels.

## Background

Colorectal cancer (CRC) is globally the third most common cause of cancer, and in the Western population approximately 5% will be diagnosed with CRC during their lifetime [1]. CRC incidence is strongly related to age, and in developing countries the median age at diagnosis is 68 years for men and 73 years for women [2]. Most CRC cases are sporadic and progress gradually over time through the adenoma-carcinoma sequence [3]. Commonly symptoms of CRC (i.e. change in bowel habits, haematochezia) are presented late in the course of the disease. However, the most important prognostic factor is the stage of the disease at diagnosis [3]; therefore screening programs for CRC have been established in many countries on subjects beyond 60 years of age [4].

The golden standard for CRC screening is colonoscopy but this has the disadvantage of being invasive, more resource demanding and expensive [5]. Instead, the method of screening for CRC used in most countries is the faecal haemoglobin (F-Hb) test followed by a colon investigation in those subjects with a positive test [4]. The accuracy of the F-Hb test depends on the method used and the cut-off value for defining a positive test [6]. The faecal immunochemical tests (FIT) perform better than the guaiac based F-Hb test, and FIT has a specificity of 94% in detecting CRC [7]. In contrast, FIT has only a modest sensitivity 74% and it fails to detect precancerous stages (polyps and adenomas) [7].

The lack of sensitivity for FIT to detect early tumor stages of CRC highlights the need to find novel biomarkers that could improve the screening detection rate for CRC. Faecal calprotectin (FC) is a marker that is currently being used to differentiate between organic and functional disorders of the colon. Calprotectin is a calcium binding protein that is abundant in the cytosol of neutrophils, and accordingly, the levels of FC will rise in faeces when active inflammation is present [8]. Previous studies have shown that patients with CRC have increased FC levels, and that FC reverts to normal levels after resection surgery [9]. The reason for why patients with CRC have elevated FC levels is not known. Possible causes are occult bleeding or tumor leakage. In addition, recruitment of neutrophils at the tumor site has been suggested. For example, a local acute inflammatory reaction of variable intensity has been seen in patients with CRC [10].

The primary aim of the present study was to determine to what extent FC levels are associated with tumor localization and tumor stage. We hypothesized that distal location, advanced stage and inflammatory activity are associated with higher FC levels. The secondary aim was to study the association between FC levels within three months prior to diagnosis and common serum biomarkers.

## Material and methods

### Study design

A retrospective, observational study of patients with CRC in clinical practice.

### Study population

**Fig. 1:**
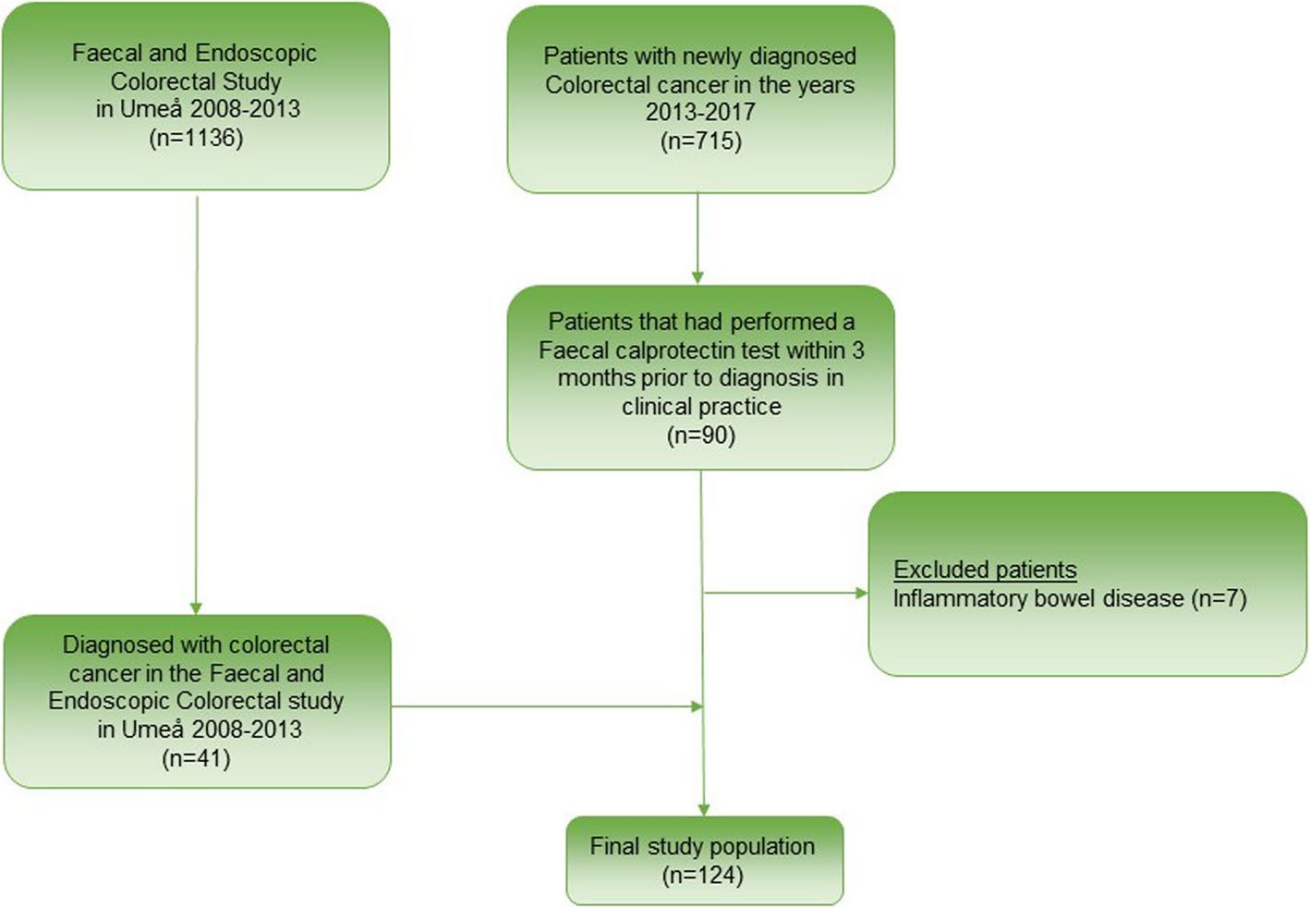
Flow chart of the selection process of patients for the study

The study included two cohorts. The first cohort included all patients between January 1, 2013 to September 14, 2017 in Västerbotten County Sweden with newly diagnosed CRC according to International Classification of Diagnosis (ICD) codes C18-21 and D37.4–5. Västerbotten County is situated in Northern Sweden and has a population of 220,000 inhabitants. There are three hospitals in the area. The entire region shares the same computerized medical record system, which enables easy access to all chemical laboratory analyses, diagnostic imaging, and all sections of the medical record (i.e. from the Department of Surgery and Department of Oncology, etc.). A medical chart review that included journals from Surgery, Endoscopy, Radiology and Pathology was performed to confirm diagnosis and inclusion/exclusion criteria. An inclusion criterion was a FC test performed within three months prior to CRC diagnosis (Fig. 1). All patients diagnosed with inflammatory bowel disease [11] were excluded from the study.

A second cohort of patients with CRC were recruited from the *Faecal and Endoscopic Colorectal study in Umeå* (FECSU); FECSU has been described in detail previously [12]. Briefly, in the FECSU study, all out-patients referred for colonoscopy from 2008 to 2013, regardless of underlying indication, were offered to undergo a FC test and a FIT on the day before the start of the bowel preparation for colonoscopy. Exclusion criteria in the FECSU were planned colonoscopy within one week, dementia and low-performance status that included mentally or physically disabled persons. In the FECSU, 1136 patients were enrolled of which 41 patients were diagnosed with CRC at the colonoscopy.

### CRC diagnosis and observational time

For all patients in the present study, the diagnostic date for CRC was defined by when the tumor was located for the first time with either endoscopy, imaging or surgery. The characteristics of the CRC, i.e. location and tumor stage according to the TNM classification [13], was defined by information from endoscopy reports, surgery reports as well as judgement from the pathologists. For those patients who received radiation therapy prior to surgery, the clinical tumor stage before the start of radiation therapy was used. A tumor located proximal of the splenic flexure was defined as right-sided, and a tumor located distally of splenic flexure to the rectum was defined as left-sided. Observational time was defined as the time period between the date at diagnosis and medical chart review date of October 2018.

### Faecal and blood markers

Patients in both cohorts received the same instructions for the collection procedure of the FC test. Stool samples were stored at room temperature for a maximum of 7 days before being processed at the lab facility. The FC analyzing method used during this period at the accredited Department of Laboratory Medicine, Clinical Chemistry, University Hospital of Umeå was CALPRO® (calprotectin ELISA test) and was performed according to the manufacturer’s instructions. Assay sensitivity for FC is between 20 µg/g and 10,000 µg/g. We used two cut-off levels to define a pathological FC test. The cut-off level of ≥ 50 µg/g for the CALPRO® that was used in the original studies by Tibble et al. [14, 15]. The cut-off level of ≥ 100 µg/g which is standard to use in clinical practice [16].

FIT was recorded as positive > 40 ng/ml of human haemoglobin, or negative. FIT was analyzed using the immunological analysis FOB test (FIT) (ANL products AB, Sweden) according to the manufacturer’s instructions.

When serum C-reactive protein (CRP), serum carcinoembryonic antigen (CEA), blood haemoglobin (Hb), mean corpuscular volume (MCV), serum ferritin and FIT tests were performed in clinical practice, the tests that were sampled in closest proximity to the performed FC sample were noted. Blood tests that were performed after treatment (i.e. surgery) of the tumor, or blood test done more than three months from the performed FC were not included in the study.

### Statistical analysis

To analyze the relationship between serum biomarkers and FC we used the Spearman correlations test. The Mann–Whitney U test was used for categorical variables. The chi^2^ test was used for cross-table analysis. Logistic regression was used for dependent variable FC ≥ 100 µg/g and FC ≥ 50 µg/g and adjusted for the independent variables gender, age, localization (right-sided versus left-sided), tumor stage (stage III/IV vs I/II) and the use of acetylsalicylic acid (ASA) prescription [17]. We adjusted only for confounders that more than 10 subjects reported. Only six patients were on non-steroidal anti-inflammatory drugs (NSAID) [18] and were therefore not included in the logistic regression model. In all analyses, a p-value ≤ 0.05 was considered statistically significant. The survival time was analyzed using the Kaplan–Meier method and Mantel-Cox test. A Cox regression analysis was performed for the covariates age at diagnosis, male gender, tumor stage III/IV, right-sided tumor localization, ASA use and FC ≥ 100 µg/g. All statistics were calculated using SPSS version 27.

## Results

**Fig. 2:**
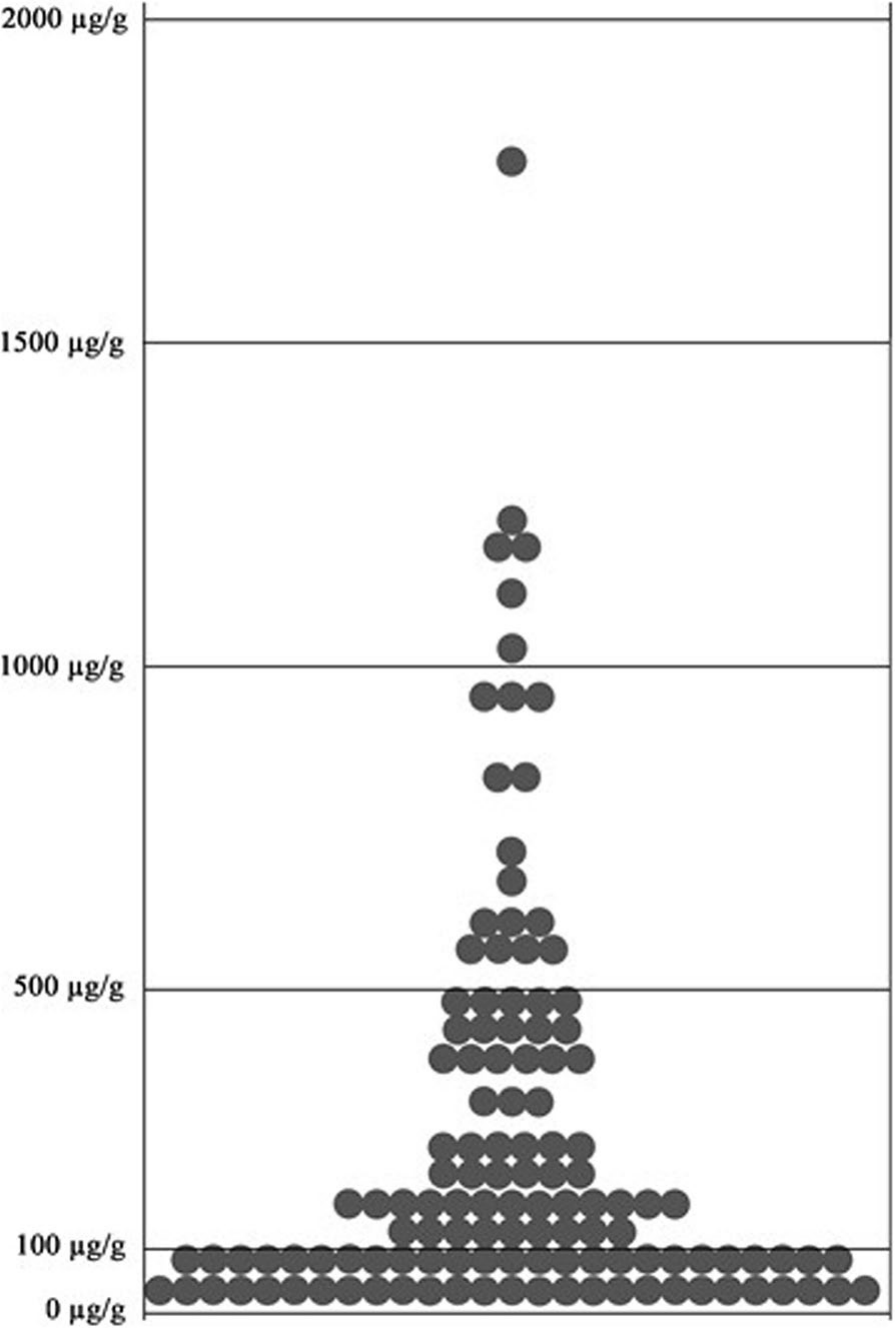
The distribution of pre-diagnostic fecal calprotectin (CALPRO®) in 124 patients with colorectal cancer

A total of 124 patients were included in the analyses (Fig. 1). Patients´ baseline characteristics are summarized in Table 1. The distribution of FC levels is shown in Fig. 2. When excluding the patients in the FECSU study (those performing the FC test the day before the preparation for colonoscopy), the median time period between FC sample and CRC diagnosis was 29 days (25^th^-75^th^ percentile 14–63 days). The frequency of concurrent medications that could have impact on FC values was acetylsalicylic acid (ASA) for 21 patients and non-steroid anti-inflammatory drug (NSAID) for 6 patients.

**Table 1:** Clinical characteristics of patients with colorectal cancer who performed a CALPRO® faecal calprotectin (FC) test within three months prior to diagnosis (*n* = 124)

Mean age, years (SD)	68 (11)
Gender	
*Women*	44% (*n* = 55)
*Men*	56% (*n* = 69)
Colorectal cancer diagnosed at:	
*Colonoscopy*	92% (*n* = 114)
*Imaging*	6% (*n* = 7)
*Surgery*	2% (*n* = 3)
Median F-calprotectin level (μg/g) (25^th^-75^th^ percentile)	149 (67–392)
Proportion of patients with:	
*F-calprotectin* ≥ *50 μg/g*	79% (*n* = 98)
*F-calprotectin* ≥ *100 μg/g*	60% (*n* = 74)
*Positive faecal immunchemical test (n* = *76)*^*a*^	89% (*n* = 68)
Tumor characteristics (*n* = 108)^a^	
*Low-grade*	85% (*n* = 92)
*High-grade*	15% (*n* = 16)
*Non-Mucinous*	85% (*n* = 92)
*Mucinous*	15% (*n* = 16)
*Vascular invasion (n* = *103)*^*a*^	18% (*n* = 19)
*Perineural growth (n* = *103)*^*a*^	19% (*n* = 20)
TNM classification	
Tumor (T) (*n* = 109)^a^	
*T1*	3% (*n* = 3)
*T2*	19% (*n* = 21)
*T3*	61% (*n* = 66)
*T4a*	9% (*n* = 10)
*T4b*	8% (*n* = 9)
Nodes (N) (*n* = 106)^a^	
*N0*	49% (*n* = 54)
*N1*	25% (*n* = 29)
*N2*	20% (*n* = 23)
Metastasis (M):	
*M0*	80% (*n* = 99)
*M1*	20% (*n* = 25)
Tumor Stage	
*I*	20% (*n* = 24)
*II*	28% (*n* = 35)
*III*	32% (*n* = 40)
*IV*	20% (*n* = 25)
Tumor localization	
*Right colon*	23% (*n* = 29)
*Transverse colon*	6% (*n* = 7)
*Left colon*	29% (*n* = 36)
*Rectum*	42% (*n* = 52)
Median values (25th-75th percentile) of:	
*C-reactive protein (mg/L) (n* = *100)*^*a*^	4.5 (1.5–13)
*B-haemoglogin (g/L) (n* = *121)*^*a*^	133 (108–142)
*Mean corpuscular volume (fL) (n* = *121)*^*a*^	88 (82–92)
*Serum carcinoembryogenic antigen (μg/L) (n* = *116)*^*a*^	3.1 (1.8–6.9)
*Serum ferritin (μg/L) (n* = *48)*^*a*^	42 (13–88)

^a^Number of subjects with available data

### Tumor localization and stage

**Fig. 3:**
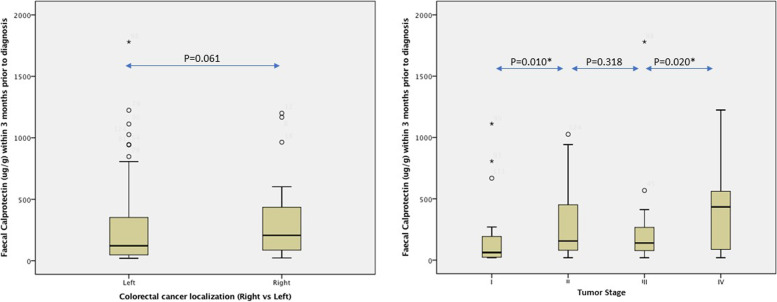
The distribution of pre-diagnostic fecal calprotectin (CALPRO®) in 124 patients with colorectal cancer according to tumor localization and tumor stage

There was no difference in tumor stage between the patients with right-sided vs left-sided CRC (56% vs 51% had tumors in stage III or IV; *p* = 0.66). Patients with right-sided tumors showed higher median FC values than patients with left-sided tumors (206 vs 122 µg/g) (Fig. 3), but the difference was not significant (*p* = 0.061). The patients with right-sided CRC had significantly more often a FC ≥ 50 µg/g than patients with left-sided CRC (92% vs 74%; *p* = 0.027). The patients with right-sided CRC also more often showed a FC ≥ 100 µg/g than the patients with left-sided CRC but the difference was not significant (72% vs 54%; *p* = 0.069).

There was no difference in the median FC values between patients with tumor stage III/IV vs patients with tumor stage I/II (Table 2), but there was a statistically significant correlation between FC levels and tumor stage (rs 0.24; *p* = 0.007). Patients with tumors in stage I had significant lower FC levels than patients with stage II (*p* = 0.010), stage III (*p* = 0.026) and stage IV (*p* = 0.005). Patients with tumors in stage IV had significantly higher FC levels than patients with tumor stage III (*p* = 0.020), but there was no difference in FC levels between patients with tumor stage II and III (*p* = 0.318) or between patients with stage II and IV (*p *= 0.248) (Fig. 3).

**Table 2:** Pre-diagnostic faecal calprotectin (FC) levels in patients with colorectal cancer (*n* = 124) in relation to tumor localization and tumor characteristics. Statistics: Mann Whitney U test

	**% (n)**	**Median F-calprotectin, μg/g (25**^**th**^**-75**^**th**^** percentile)**	***p*-value**
Tumor localization			
*Left-sided colorectal cancer*	72% (*n* = 88)	122 (48–380)	0.061
*Right-sided colorectal cancer*	28% (*n* = 36)	206 (86–435)	
Tumor stage			
*I-II*	48% (*n* = 59)	122 (32–392)	0.157
*III-IV*	52% (*n* = 65)	172 (86–412)	
Tumor grade			
*Low-grade*	85% (*n* = 92)	150 (75–374)	0.647
*High-grade*	15% (*n* = 16)	201 (73–480)	
Other tumor characteristics			
*Tumor type*			
*Non-Mucinous*	85% (*n* = 92)	146 (73–227)	**0.006**
*Mucinous*	15% (*n* = 16)	457 (122–596)	
* Vascular invasion:*			
*No*	85% (*n* = 84)	168 (77–392)	0.395
*Yes*	18% (*n* = 19)	94 (74–312)	
* Perineural growth*			
*No*	81% (*n* = 83)	150 (75–341)	0.742
*Yes*	19% (*n* = 20)	159 (74–424)	

Patients with mucinous tumors showed significantly higher FC levels compared to patients with non-mucinous tumors. There was no difference in FC levels between patients in regard of tumor grade, vascular invasion and perineural growth (Table 2).

### The association of FC and serum markers

A moderate but statistically significant correlation was seen between FC levels and serum CRP levels (rs 0.31; *p* = 0.001). Furthermore, a significant inverse correlation was seen between FC levels and B-haemoglobin (rs -0.21; *p* = 0.019) and a significant inverse correlation was seen between FC levels and MCV (rs -0.30; *p* = 0.001). There were no significant correlations between FC levels and serum CEA (rs 0.12; *p* = 0.17) and serum ferritin (rs 0.045; *p* = 0.63).

### FC levels and survival

**Fig. 4:**
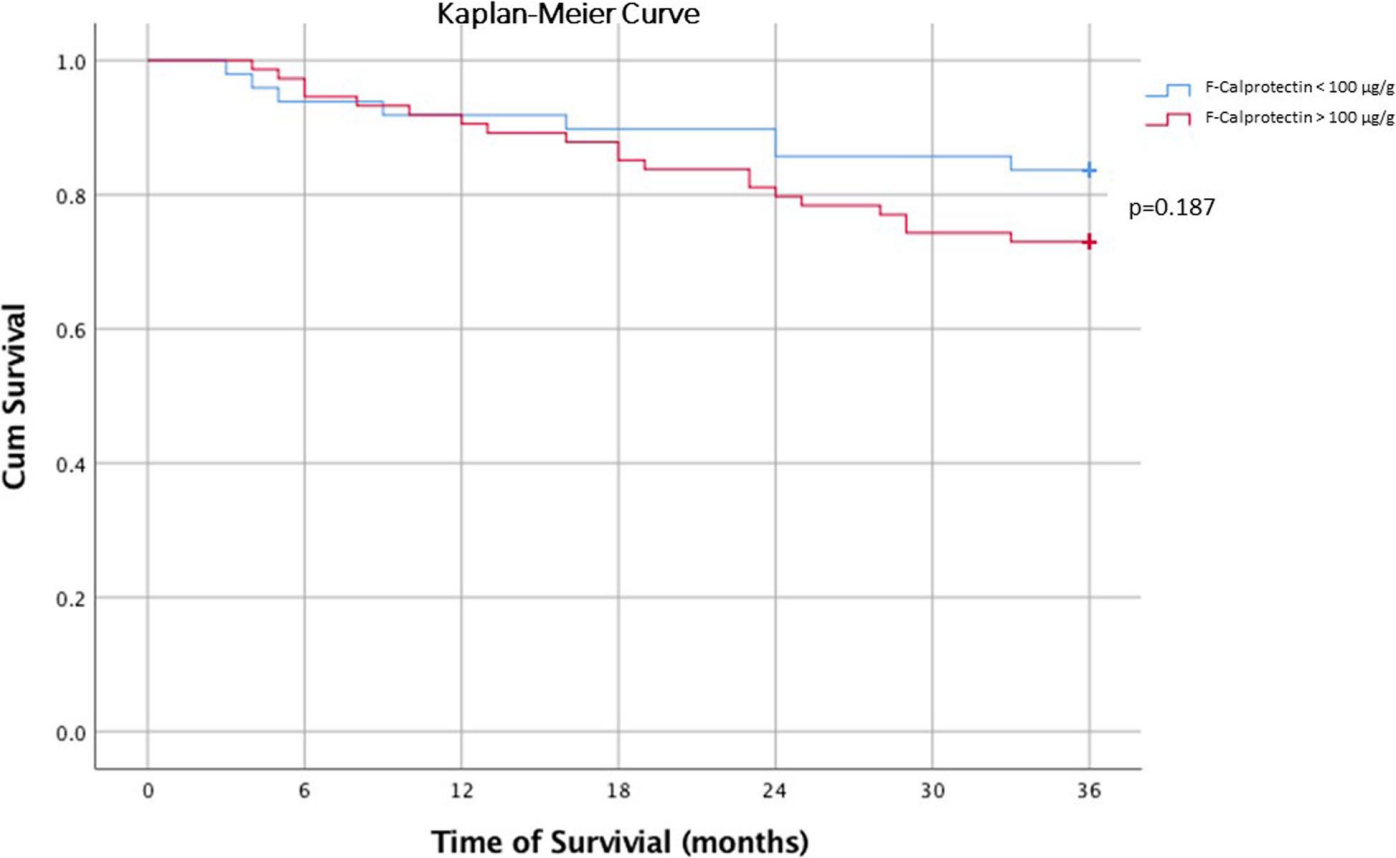
The survival of patients with colorectal cancer (*n* = 124) according to pre-diagnostic faecal calprotectin levels (Kaplan–Meier)

Twenty-eight patients (23%) deceased within 36 months. There were no significant differences in survival time between patients with FC ≥ 100 µg/g versus patients with FC ≤ 100 µg/g (Fig. 4). In a Cox regression analysis with the covariates: age at diagnosis, male gender, tumor stage (III/IV vs I/II), tumor localization (right vs left), ASA use and FC ≥ 100 µg/g, age at diagnosis (HR 1.09; CI 1.03–1.15), tumor stage III/IV (HR 69.6; CI 8.66–560) and right-sided tumor localization (HR 3.01; CI 1.31–6.92) were associated with mortality. FC ≥ 100 µg/g showed no association with survival (HR 0.61; CI 0.24–1.52) in the Cox regression analysis.

### Logistic regression

To adjust for possible confounders, we performed a logistic regression with FC as a dependent variable, and age, gender, tumor localization (right vs left-sided), tumor stage (III/IV vs I/II) and ASA use as independent variables. In the multivariate analysis for FC ≥ 50 µg/g, tumor stage and right-sided localization were significantly associated with FC. Using FC ≥ 100 µg/g as dependent variable, tumor stage and the use of ASA was significantly associated with FC (Table 3). Excluding the six patients with NSAID did not change the Hazard Ratios for tumor localization and tumor stage.

**Table 3:** Logistic regression with pre-diagnostic faecal calprotectin (FC) ≥ 50 μg/g and ≥ 100 μg/g as a dependent variable adjusted for age, gender, tumor localization, tumor stage, and the use of acetylsalicylic acid in patients with colorectal cancer (*n* = 124). OR = Odds Ratio

	**Patients with F-calprotectin ≥ 50 μg/g (*n* = 98)**		**Patients with F-calprotectin ≥ 100 μg/g (*n* = 74)**	
	Univariate OR (95^th^ confidence interval)	Multivariate OR (95^th^ confidence interval)	Univariate OR (95^th^ confidence interval)	Multivariate OR (95^th^ confidence interval)
Age	1.03 (0.99–1.08) *p* = 0.118	1.02 (0.98–1.08) *p* = 0.261	1.02 (0.99–1.07) *p* = 0.114	1.02 (0.98–1.07) *p* = 0.248
Male gender	1.62 (0.68–3.87) *p* = 0.276	1.65 (0.64–4.27) *p* = 0.297	0.98 (0.47–2.01) *p* = 0.948	0.91 (0.41–1.97) *p* = 0.804
Right-sided vs Left-sided colorectal cancer	**3.89 (1.08–14.0)** ***p* = 0.037**	**3.80 (1.01–14.3)** ***p* = 0.048**	2.17 (0.93–5.03) *p* = 0.072	2.05 (0.85–4.94) *p* = 0.108
Tumor stage III/IV vs I/II	**2.52 (1.02–6.21)** ***p* = 0.045**	**3.47 (1.27–9.42)** ***p* = 0.015**	1.76 (0.85–3.65) *p* = 0.124	**2.30 (1.04–5.10)** ***p* = 0.039**
The use of acetylsalicylic acid	6.41 (0.81–50.3) *p* = 0.077	7.30 (0.86–62.2) *p* = 0.069	**3.43 (1.07–10.9)** ***p* = 0.037**	**3.54 (1.03–12.2)** ***p* = 0.045**

## Discussion

The aim of the present study was to investigate how the tumor characteristics are associated with pre-diagnostic FC levels in patients with CRC. In line with previous studies [9, 19–22] we found a high prevalence of elevated FC levels in patients with CRC, and 4 out of 5 patients showed a FC ≥ 50 µg/g just prior to diagnosis.

A positive correlation between FC levels and the degree of tumor growth (TNM stage) was shown in previous studies [9, 10], and the present study confirms in a large cohort that patients with CRC at more advanced stages have higher FC levels. To our knowledge, this is the first study that also showed a moderate significant association between the proximal location of CRC and FC levels. Nine out of 10 patients with right-sided CRC had a FC ≥ 50 µg/g, which was significantly more common than in patients with left-sided CRC. We found only one published report on FC levels that included information on tumor location [19]. When combining the patients included in that previous study with our data, patients with right-sided CRC still show significantly higher FC levels than patients with left-sided CRC (median 214 µg/g vs 129 µg/g; *p* = 0.03).

It is known that right-sided CRC is more clinically quiescent than, for example rectal cancers, and consequently presents with symptoms at a more advanced stage than distal tumors [23]. However, when using a logistic regression model, adjusting for tumor stage, right-sided CRC location still shows a significant association with increased FC levels. In a recent review by Baran et al. [24], the differences between left-sided and right-sided CRC were investigated. The authors stated that there are differences between right-sided CRC and left-sided CRC in embryological origin, tumor histology and therapy response. Right-sided CRC was predominately characterized by high microsatellite instability and mutations in *KRAS* or *BRAF* in comparison to the left-sided tumors that instead showed a higher chromosomal instability. The high microsatellite instable tumors were shown to carry more immunogenic mutation and histologically resembled a lymphoid reaction similar to that seen in Crohn´s disease. Previous research has argued that the most likely mechanism for increased FC levels in patients with CRC is an increased permeability of the tumor pathologic mucosa membrane that leads to high amounts of neutrophils in the gut lumen. Another contributing factor to FC levels in patients with CRC could be an effect of chemotactic stimuli from the tumor that recruits granulocytes containing calprotectin [25]. For example, calprotectin has been shown to reduce tumor cell growth by inducing apoptosis of the cancerous cells [26].

Perhaps, a tumor located on the right-sided colon shows a more immunogenic tumor characteristics than a left-sided tumor and therefore presents with a higher FC level. If this is so, adding a FC test to CRC screening programs could improve the detection of right-sided CRCs. Interestingly, the patients in our study showed a positive correlation between serum CRP levels and FC levels; this may reflect increased inflammatory activity at the tumor site. However, in a study by Lehman et al., there was no correlation between histological inflammation at the tumor and FC levels [10].

Calprotectin in the stool has its source mainly from the leakage of white blood cells into the lumen. Therefore, bleeding from the tumor could also be a cause of an elevated FC level in a patient with CRC. A positive FIT was common in the patients in our study, and both B-haemoglobin and MCV inversely correlated to FC levels in our patients. It is reasonable to presume that a tumor that has reached a more advanced stage is also easier to bleed. But on the other hand, it would require a large volume of blood to produce significant increases in FC levels, which indicates that factors other than bleeding contribute to the rise in FC levels in patients with CRC [27].

The present study has some limitations. The main limitation is that the study included only approximately 10% of all patients with a CRC diagnosed during the study period. For example, in the cohort of patients recruited 2013 to 2017, the doctor´s decision to test for FC in most cases was probably due to the investigation of diarrhea with other diagnoses other than CRC in focus. The slightly higher proportion than expected of patients with rectal cancer in our study might have been due to rectal cancers that affect defecation more than for other CRCs. GI symptoms often present late when the tumor has reached a more advanced stage.

Therefore, inflammatory activity and bleeding from the tumor has become obscure in symptomatic patients. A prospective study that includes subjects in a CRC screening program is suggested to test the accuracy of FC for earlier stages of CRC. Finally, there could be other confounders that were not included (i.e. drugs) that could have influenced the results. Nonetheless, to our knowledge, our study presents the largest data set on FC levels for patients with CRC.

To conclude, FC levels correlate with tumor stage and to proximal CRC location. Tumor stage shows the strongest association with an increased FC level. In clinical practice, the role of FC in the context to differentiate between tumor stage or tumor localization is probably less important. However, our results confirm that elevated FC levels are common in patients with CRC and doctors should be aware that even minor elevation of FC could be associated with CRC. Also, our findings implicate that FC could be used as a possible marker in combination with other markers in the screening of CRC, especially for right-sided tumors.

## Data Availability

The datasets generated and/or analysed during the current study are not publicly available due Swedish law but are available from the corresponding author on reasonable request.
